# Concerns about COVID-19 Vaccine Hesitancy among Law Enforcement Officers: Prevalence and Risk Factor Data from a Nationally Representative Sample in the United States

**DOI:** 10.3390/vaccines11040783

**Published:** 2023-03-31

**Authors:** Bruce G. Taylor, Elizabeth A. Mumford, Alejandra M. Kaplan, Weiwei Liu

**Affiliations:** NORC at the University of Chicago, Chicago, IL 60603, USA

**Keywords:** law enforcement officers, COVID-19 vaccine hesitancy, COVID-19 pandemic, national survey, probability-based sample, officer health

## Abstract

Scant research exists on COVID-19 vaccine hesitancy among law enforcement officers, hindering health messaging development for officers and, by extension, the communities they serve. This paper’s goal was to address this gap by providing the necessary data to better under hesitancy to guide training and policy interventions for officers. The objective was to conduct the first nationally representative survey of officers on COVID-19 vaccine hesitancy and its correlates. We collected data from February 2021 to March 2022 on officer COVID-19 vaccine hesitancy and examined their responses in terms of sociodemographic factors, health status, and job characteristics. We found that 40% of officers were COVID-19 vaccine hesitant. We found that officers with higher education, older officers, officers with more law enforcement experience, officers who received recent health checkups, and commanders (compared to line officers) were less likely to be COVID-19 vaccine hesitant. Critically, officers working in law enforcement agencies that provided masks for COVID-19 protection were less likely to be COVID-19 vaccine hesitant (compared to agencies not providing masks). Ongoing research is needed to understand how evolving attitudes and barriers toward vaccination change over time for officers and to test messaging to better align officers with health guidelines.

## 1. Introduction

As of January 2023, there are over 100 million SARS-CoV-2 (COVID-19) cases in the US and over one million deaths attributed to COVID-19 [1]. With the acceleration of the COVID-19 vaccine program nationwide in the US reaching two-thirds of the United States’ adult population with at least one dose by July 2021, significant reductions in symptomatic infections, hospitalization, and mortality caused by the virus were observed [2,3,4]. However, higher vaccination coverage rates are necessary to enable greater indirect protection for the overall community, return society to a normal pattern of life, and rejuvenate the economy [5]. While safe and effective vaccines for COVID-19 have been made readily available for free in the US, we have still yet to achieve the widespread uptake that is necessary to more significantly reduce and control COVID-19 morbidity and mortality and to achieve herd immunity [6]. That is, while about 80% of the US population has had one dose of the COVID-19 vaccine by January 2023, only 15.4% of them are up to date on their COVID-19 booster vaccines [1].

Vaccine hesitancy, which refers to the delay in acceptance or refusal of vaccination despite the availability of vaccination services [7], has been documented as a key barrier to the success of the COVID-19 immunization programs in the US [8]. While there has been extensive research on COVID-19 vaccine hesitancy with the US general population [8] and among certain workgroups, such as healthcare workers [9], we know little about COVID-19 vaccine hesitancy among law enforcement officers (LEOs), the focus of this paper. In the US context, LEOs are public-sector employees whose sworn duties are to enforce the laws, maintain public order, and manage public safety. In this study, we focused on municipal-, county-, and state-level officers who primarily are patrol officers, detectives, sheriff deputies, or state troopers (federal law enforcement is not included in this study). It is especially crucial to understand COVID-19 vaccine hesitancy among LEOs because LEOs, as frontline workers, have had high exposure to COVID-19 throughout the pandemic, continue to be very vulnerable to contracting COVID-19 by virtue of the nature of the work, and are a key workforce for maintaining the safety of the public.

### 1.1. COVID-19 Vaccine Hesitancy

Based on data from nationally representative studies, overall COVID-19 vaccine hesitancy rates range from 15.6% to 46.4% [8]. Widespread COVID-19 vaccine hesitancy has been observed throughout the pandemic, and intentions toward vaccination and reasons for vaccine hesitancy have varied over time and across communities [10]. While it is likely that COVID-19 vaccine hesitancy may be influenced by similar factors that influence hesitancy toward other types of vaccines [11], COVID-19 is unique in modern times in its novelty and severity, its accompanying widespread disruption of everyday life, the polarized political climate in which the pandemic has unfolded, and the historic speed of the development of the COVID-19 vaccine.

The reasons for COVID-19 vaccine hesitancy are varied and include concerns over side effects and efficacy of the vaccine; fears over government abuse of power; and even extreme views, such as covert cell-based microchip implantation [12]. Moreover, vaccine hesitancy is not a new obstacle for disease prevention, for it has been a significant barrier for addressing seasonal influenza and the 2009 H1N1 pandemic [13,14], and it is part of a social movement toward vaccine opposition that has been growing in the US [15].

To better understand population responses to vaccination, a large number of studies have examined relationships between sociodemographic, behavioral, and attitudinal factors and COVID-19 vaccine hesitancy [16,17,18,19]. Understanding factors associated with and reasons for COVID-19 vaccine hesitancy is essential to tailor public health messaging and to develop interventions that address patient concerns while maximizing vaccine uptake. In this study, we identified sociodemographic, health status, job characteristics, and job-site factors associated with vaccine hesitancy among law enforcement officers to better understand how to focus vaccination messaging interventions for this population.

Individual (demographics, health history, behaviors, and health beliefs), interpersonal (having a close friend/family member impacted by COVID-19), healthcare, and societal-level factors (healthcare provider recommendations, source/credential of COVID-19 related information, and COVID-19-related conspiracy theories) all contribute to COVID-19 vaccine hesitancy in the US [8]. A number of studies in the US identified a number of key factors associated with COVID-19 vaccine hesitancy. These factors include the following: minority ethnic group status (those of Hispanic or non-Hispanic Black ethnicity are more vaccine hesitant), gender (women are more vaccine hesitant), age (younger people are more hesitant, partially as they perceived being at lower risk compared to older persons for the COVID-19 virus), educational attainment (participants with less than a college degree are more vaccine hesitant), religious considerations (fear that the origin of the vaccine is related to aborted fetal tissue), relative experience of COVID-19 infection among members in their home or community, community norms (e.g., participants living in a rural area are more COVID-19 vaccine hesitant), media misinformation (e.g., conspiracies on social media), and non-use of healthcare resources [5,8,20,21,22,23,24]. Moreover, some studies have found that COVID-19-related anxiety and adverse mental health conditions (e.g., anxieties around COVID-19) are related to COVID-19 vaccine hesitancy [25,26,27,28].

### 1.2. COVID-19 Vaccine Hesitancy among Law Enforcement Officers

During the COVID-19 pandemic, police officers have been responsible not only for enforcing the law and protecting our communities but also playing a role in controlling the spread of COVID-19, including enforcing lockdowns, travel bans, and social-distancing measures [29]. As LEOs are first responders, they have been, and continue to be, on the front lines for every variant of the coronavirus as it has been actively spreading, leaving LEOs highly vulnerable to contracting COVID-19. While there are no specific data in the literature on COVID-19 positivity rates for LEOs as a group, officer deaths associated with COVID-19 have been tracked. COVID-19 was responsible for a large proportion of all officer in-duty deaths from 2020 to 2022 according to the Officer Down Memorial Page, a database that tracks line of duty officer deaths. In 2020, out of 436 in-duty officer deaths, more than half (282) were from COVID-19 [30]; in 2021 over 70% (474) of the 669 total officer in-duty deaths were due to COVID-19 [31]; and the rates were somewhat lower in 2022, with about one-third of officer in-duty deaths due to COVID-19 (73/229) [32].

While rates of COVID-19 vaccine hesitancy have been noted to be high among some first responders, such as healthcare workers and emergency medical service (EMS) professionals in some settings compared to the general population [9,33,34], we know little about COVID-19 vaccine hesitancy rates among law enforcement officers, thus hindering policy development around COVID-19 for this group. Using a convenient sample of a police magazine readers, 3328 officers, police administrators, police leaders and police trainers completed a brief survey in December 2020 on their willingness to receive a COVID-19 vaccine [35]. About 51% of these respondents reported that they would get the vaccine, and 49% expressed vaccine hesitancy (38% said they will not be vaccinated for COVID-19, and another 11% were unsure) [35]. While there are differences among correctional officers and LEOs, one small study of correctional officers in a single Massachusetts jail found that only 18% were COVID-19 vaccine hesitant [36].

Our study addresses several gaps in the literature on COVID-19 vaccine hesitancy. First, we are studied COVID-19 vaccine hesitancy for a group of first responders, LEOs, for whom almost no data exist on this topic. The single study published regarding this topic among US LEOs relied on a convenience sample of unknown reliability, whereas our study applied a national probability-based representative sampling approach. The absence of data on COVID-19 vaccine hesitancy among officers is an important gap in the literature. Second, LEOs have frequent contact with the general public and could potentially spread COVID-19 through their high levels of contact with others in the community. Moreover, some members of the public look to officers as role models and community heroes; officers’ expressed vaccine hesitancy could lead others in the community to be hesitant regarding COVID-19 vaccines. Third, this is the first study with LEOs to examine correlates of COVID-19 vaccine hesitancy. Our study used multivariate modeling to examine a range of correlates found to be important in examining COVID-19 vaccine hesitancy and potentially informing public health and law enforcement communication messages for future emergencies. However, as noted in the Section 5, not all the important variables from the literature are included in our study.

### 1.3. The Present Study

Our team conducted the first, to our knowledge, nationally representative sample survey of LEOs during the COVID-19 pandemic as part of a larger project called the Officer Safety and Wellness (OSAW) Initiative. Given the nascent stage of research regarding law enforcement officers and COVID-19 hesitancy, this was an exploratory study. We asked LEOs about their hesitancy toward receiving the COVID-19 vaccine. The study examined sociodemographic, relationship/marital status, health status, job characteristics, and whether the agency provided masks as factors associated with vaccine hesitancy. These factors were examined to better understand how to focus public health messaging around COVID-19 vaccines for LEOs.

Our study design provides data to answer our first research question: What is the extent of COVID-19 vaccine hesitancy reported by LEOs in the US? Based on prior research on vaccine hesitancy for the general population [5,8] and descriptive data from some small exploratory studies on COVID-19 vaccine hesitancy for LEOs [35,36], we expected LEOs to have greater hesitancy toward COVID-19 vaccines compared to the general population. Our second research question relates to the relationship between LEOs’ health status (receiving health checkups, using services of a medical professional, and emotional wellness status) and COVID-19 vaccine hesitancy. We assessed if those who take better care of their health through health checkups and/or seeing a medical professional and who report higher levels of emotional wellness were less likely to be COVID-19 vaccine hesitant. Our third research question investigated the relationship between job characteristics (officer rank and years working as an officer) and the approach to COVID-19 at the job site (whether the agency provided masks) and LEOs’ COVID-19 vaccine hesitancy. Without prior research in this area, we were uncertain about whether aspects of the LEO work environment, particularly around COVID-19, would be related to COVID-19 vaccine hesitancy among LEOs. Despite the lack of prior research, LEOs’ work environment is an important area to investigate because of the possible policy implications for agency administrators.

## 2. Materials and Methods

### 2.1. Ethical Considerations

We received Institutional Review Board (IRB) approval from the authors’ institution that holds a multiple project assurance with Health and Human Services (HHS) and conforms to HHS standards for IRB review. Informed consent was obtained from all study participants prior to their starting a survey.

### 2.2. Procedures

Our team used a two-stage probability-based sampling approach, a common approach in developing a nationally representative samples of officers [37], for the OSAW Initiative. First, for our Stage 1 sample, collected from August 2017 to February 2019, we started with the National Directory of Law Enforcement Administrators (NDLEA), Correctional Institutions and Related Agencies (53rd Edition) [38]. We used the NDLEA to randomly select a nationally representative sample (*n* = 1135) of publicly funded civilian law enforcement agencies (LEAs), inclusive of city, county, state police, highway patrol, and Native American/tribal LEAs. We did not include federal LEAs in the study. Moreover, all LEAs included in the study needed to have at least one full-time sworn LEO with arrest powers. We collected the data for this study on COVID-19 vaccine hesitancy during the third wave of OSAW data collection. We conducted the survey within the first year of COVID-19 being declared a national emergency from February 2021 (a time when COVID-19 deaths had reached 500,000 and about 46.1 million people, or 13.9%, of the US population had received at least one dose of the COVID-19 vaccine [39]) to March 2022 (when the number of recorded deaths due to COVID-19 had reached 976,229 and 255 million people, or 76.8%, of the total US population had received at least one dose of the vaccine) [40]. It is also worth noting that, in November 2021, about nine months into our 14-month data collection period, the COVID Omicron Variant was first named by the World Health Organization (WHO) and had become the dominant strain in the US from late December 2021 to early 2022 [41].

In Stage 2, we selected representative random samples of LEOs from rosters of officers produced from a stratified random subsample of participating LEAs (*n* = 140). The Stage 2 sampling was designed to select a group of LEOs from each roster based on the number of officers in each agency, while also oversampling females who are underrepresented in LEAs at a rate of 2:1 [42]. We used multiple methods and multiple reminder prompts (often up to 12 attempts) through emails, phone calls, and reminder mailings sent via the US Post Office to reach participants and encourage participation. Unless we received a refusal to participate, the survey-reminder prompts continued to provide officers with the opportunity to participate in the survey. Due to complications with some agencies’ policies, no monetary incentives or incentives other than contributing to the science of officer wellness were offered for participation. To assure confidentiality to LEO participants, we did not inform the participating LEAs as to which LEOs participated in the survey. Of the 3330 officers eligible to participate in the third wave of the OSAW Initiative, 1050 officers submitted completed surveys, for a response rate of 32%, a response rate better than achieved in other established panels in other fields [43]. A comparison of the 2280 participants to 1050 non-participants revealed no statistically significant differences on key demographic and background factors, thus suggesting that our 32% response rate did not introduce bias. Most of the participating officers completed all the survey questions, with little item-level missing values (about 5%). However, when accounting for missing data, our analytic minimum sample size is 997 officers. That is, 997 cases were available for our multivariate analyses.

All results presented are weighted to make the data nationally representative based on aggregate statistics on the gender, age, and ethnicity of the officers. Weights were calculated as the inverse of the probabilities of selection and adjusted for survey non-response [44]. We developed poststratification weights by matching the weighted sample cell counts to the population cell counts and applying a proportional adjustment to the weights. Our non-response weights took into account selection probabilities and addressed possible non-response bias with non-response adjustments. All surveys were completed via a user-friendly secure online survey that took about 30 min to complete.

### 2.3. Participants

The median age for the sample of officers was 45, with 75% of the sample being male officers and 25% female officers (see Table 1). Over 80% of the sample was non-Hispanic White, more than half (52%) were college graduates or higher, and 83% were married or living with a partner. Two-thirds (65.1%) of this sample of 1000 officers worked in municipal law enforcement, about one in four (24.1%) worked for county law enforcement, 10% worked for state police or highway patrols, and fewer than 1% worked for tribal LEAs.

### 2.4. Measures

#### Dependent Variable

1. COVID-19 Vaccine Hesitancy

To measure hesitancy to the COVID-19 vaccine, we used a brief tested tool used in other national surveys on COVID-19 vaccine hesitancy [17]. We conceptualized vaccine hesitancy as a behavior, and our measure is broadly aligned with the SAGE Working Group definition of vaccine hesitancy to include a “delay in acceptance or refusal of vaccination despite availability of vaccination services” [7].

We asked participants first, “Have you ever received a COVID-19 vaccination?” If they said “no” to receiving a COVID-19 vaccine, we asked them another question: “Now that a vaccine against the coronavirus is becoming available, do you plan to get vaccinated, or not?” The response choices were the following: “1 = Yes, I will get a coronavirus vaccine as soon as it is available to me; 2 = Yes, I will get a coronavirus vaccine, but I will wait until it is proven to be safe and effective, 3 = No, I will not get a coronavirus vaccine, and 4 = Not sure”. Supporting our modeling approach (see Analytic Plan below), we created two outcome measures. For our dichotomous outcome variable, all respondents who said that they had already received a COVID-19 vaccine or indicated that they would get a coronavirus vaccine as soon as it was available to them were coded as “0 = No, not hesitant to COVID-19 vaccine”. Next, we coded respondents 1 if they had not received the COVID-19 vaccine and answered the follow-up question as a 2, 3, or 4. For our 4-category nominal outcome of COVID-19 vaccine hesitancy, we coded as a 1 all respondents who said that they had already received a COVID-19 vaccine or indicated the following: “Yes, I will get a coronavirus vaccine as soon as it is available to me”. Next, those who responded “yes, will get vaccinated when it is proven safe and effective” were coded as a 2, “will not get vaccinated” were coded as a 3, and “unsure about getting vaccinated” were coded as a 4. This 4-category nominal outcome supports the examination of each of these hesitant groups separately compared to the reference category of not hesitant (already received a COVID-19 vaccine or would get a coronavirus vaccine as soon as it became available to them).

2. Covariates

Sociodemographic measures included age (a continuous measure), biological sex (female coded as one and male coded as zero), race/ethnicity (White (reference category), non-Hispanic Black, Hispanic, or other race) and educational attainment (bachelor’s degree or more coded 1; 0 otherwise). An indicator of living with a romantic partner was coded as zero if the LEO reported a co-resident partner (married/remarried/lived together with a partner) and one otherwise for not cohabitating (widowed/divorced/separated/never married/in a relationship, but not living together). We measured health risk status by noting if the participants had not gone for a health checkup in the past two years (no health checkups were coded as a 1, and 0 was receiving a health checkup) or not seen a medical professional for health services in the past year (coded as a 1 if they have seen a medical professional or 0 if they have not). We measured emotional wellness by using the Mental Health Inventory (MHI-5). The MHI-5 is a five-item validated scale assessing anxiety and depression [45], and it was a continuous measure (Cronbach’s alpha = 0.83; range from 0 to 100) assessed for the past month, with higher scores representing greater emotional distress.

We measured three aspects of job characteristics. We measured officer rank by using a four-level categorical variable (line officer as the reference, supervisor, commander, or other rank). The number of years of sworn LEO experience was coded as a continuous measure of years of service. The urbanicity of the policing sector in which the LEO is employed was coded as a five-level variable (only urban area (reference category), only suburban, only rural, mix of areas, and other area). We also asked about whether the agency provided face masks (“Has your agency provided you with sufficient masks to protect against coronavirus while on the job, as an officer?”). Our response options were the following: “1 = Yes—since the outset of the pandemic [reference category]”, “2 = No—masks available after initial shortage or sporadically from agency”. and “3 = No—sufficient masks never provided by agency”. We also asked officer participants if they were exposed to COVID-19 on the job.

### 2.5. Analytic Plan

Statistical analyses were conducted using IBM SPSS software version 28.0 [46]. For most of the variables, the level of item-level missing data was minimal (about 5%). Overall, most of the officers who responded to the survey skipped very few questions, so we used listwise deletion of data. First, we provide overall descriptive statistics on all of the study variables in Table 1. Table 2 examines the same variables but for the subgroups of whether the participant was or was not COVID-19 vaccine hesitant (including a chi-square test for the categorical variables of whether the difference between the two groups was statistically significant or F test for the continuous measures). We conducted two types of regression analysis: (1) binomial logistic regression to compare those who did not display any vaccine hesitancy with those who displayed some vaccine hesitancy; and (2) multinomial regression to compare those who did not display any vaccine hesitancy with the three groups of officers with various degrees of vaccine hesitancy separately, as a sensitivity analysis (albeit with some sample size constraints).

Table 3 includes the binomial logistic regression results for our dichotomous dependent variable on COVID-19 vaccine hesitancy and a set of covariates. Table 4 includes the multinomial regression results for our four-level categorical dependent variable on COVID-19 vaccine hesitancy and a set of covariates. Due to the smaller sample size of the vaccine-hesitant Group 2 (will get vaccinated when it is proven safe and effective, *n* = 79) and of the vaccine-hesitant Group 4 (unsure about getting vaccinated, *n* = 82) and the corresponding reduction of statistical power, this is more of an exploratory and sensitivity analysis to assess if the binomial logistic regression model results in Table 3 vary within our three vaccine-hesitant groups.

## 3. Results

We observed that 40.3% of officer participants were COVID-19 vaccine hesitant (see Table 1). Of officers who were COVID-19 vaccine hesitant (see Table 1), we found that 79 participants (7.4%) said that they would get vaccinated when it was proven safe and effective, 272 (25.3%) said that they will not get vaccinated, and 82 (7.6%) said that they were unsure about getting vaccinated (latter numbers not shown in the tables). Of the 59.7% (*n* = 641) who were not hesitant (see Table 1), we found that 589 participants were already vaccinated (54.8%), and 52 participants said that they would get vaccinated when it became available (4.9%) (the later numbers not in the tables). Most officers (81%) in our sample were provided masks over the study period, and 50% of the officer participants confirmed that they were exposed to COVID-19 on the job.

The vast majority of officers (81%) had received health checkups over the past two years and had seen a medical professional 3.9 times on average in the past year. More than half of the officers in our study were experiencing a common mental health disorder (depression or anxiety), scoring, on average, 72.8 out of 100 on the MHI-5 (with higher scores representing greater emotional distress). On average, our sample had 18 years of law enforcement experience (with only 20% of the sample with five or fewer years of experience), and most of the participants in the sample were either line officers (57%) or supervisors (20%). About 44% of the officers worked in urban areas, 19% in suburban areas, 16% in rural areas, and the remaining 21% in mixed or other areas.

We did observe any differences between officers who were vaccine hesitant compared to those who were not vaccine hesitant in regard to education levels, officer rank, and number of times an officer has seen a medical professional in the past year (Table 2). However, these were bivariate differences and do not control for other factors that might explain the differences between those officers who were vaccine hesitant and those who were not hesitant in regard to the COVID-19 vaccine. This leads us to our multivariate logistic regression model in Table 3.

We observed four variables that were statistically related to officers’ COVID-19 vaccine hesitancy (Table 3). Officers who were commanders in rank were 41.7% (1—0.583 Adjusted Odds Ratio or AOR) less likely to be COVID-19 vaccine hesitant compared to line officers (AOR = 0.583, *p* < 0.01). Officers working in agencies that did not provide masks for COVID-19 protection were 2.354 (AOR = 3.354) times more likely to be COVID-19 vaccine hesitant compared to officers working in agencies that regularly provided masks for COVID-19 protection (AOR = 3.354, *p* < 0.05). Officers with a college degree or higher education were 47.2% less likely to be COVID-19 vaccine hesitant compared to officers without a college education (AOR = 0.528, *p* < 0.001). The older the officer, the less likely he or she was to be COVID-19 vaccine hesitant (AOR = 0.957, *p* < 0.001).

We observed a number of variables that were statistically related to one of three categories of COVID-19 vaccine hesitancy when compared to the reference category of not being COVID-19 vaccine hesitant (Table 4). Starting with Group 2 (officers who planned to get the COVID-19 vaccine when it was proven safe/effective), we observed that officers who did not report having had a health checkup in the past two years were 1.5 times more likely to be in this hesitant group of waiting for the COVID-19 vaccine to be proven safe/effective than to be in the not hesitant group (AOR = 2.469, *p* < 0.01). Officers working in a mixed sector of urban, suburban, and rural areas were 1.25 times more likely to be in this hesitant group of waiting for the COVID-19 vaccine to be proven safe/effective than to be in the not-hesitant group (AOR = 2.246, *p* < 0.05). Officers with a college degree or higher education were 46.1% less likely to be in this hesitant group of waiting for the COVID-19 vaccine to be proven safe/effective than to be in the not-hesitant group (AOR = 0.539, *p* < 0.05). Older officers were 7.8% less likely to be in this hesitant group of waiting for the COVID-19 vaccine to be proven safe/effective than to be in the not-hesitant group (AOR = 0.922, *p* < 0.01).

Moving to Group 3 (officers who did not plan to get a COVID-19 vaccine), we observed that officers at the commander rank were 38.9% less likely to be in this hesitant group than to be in the not-hesitant group (AOR = 0.611, *p* < 0.05). Officers working for a law enforcement agency that did not provide masks were 3.12 times more likely to be in this hesitant group than to be in the not-hesitant group (AOR = 4.117, *p* < 0.05). Officers with a college degree or higher education were 38.0% less likely to be in this hesitant group than to be in the not-hesitant group (AOR = 0.620, *p* < 0.01). Older officers were 4.2% less likely to be in this hesitant group of not willing to get the COVID-19 vaccine than to be in the not-hesitant group (AOR = 0.958, *p* < 0.01).

Moving to Group 4 (officers who were not sure about getting the COVID-19 vaccine), we observed that non-Hispanic Black officers (compared to non-Hispanic White officers) were 1.76 times more likely to be in this hesitant group of “unsure” than to be in the not-hesitant group (AOR = 2.757, *p* < 0.05). Officers working in an “other” sector were 4.07 times more likely to be in this hesitant group of “unsure” than to be in the not-hesitant group (AOR = 5.067, *p* < 0.001). Officers with a college degree or higher education were 69.5% less likely to be in this hesitant group of “unsure” than to be in the not-hesitant group (AOR = 0.305, *p* < 0.001). Officers with more years as an officer were 5.3% less likely to be in this hesitant group of “unsure” than to be in the not-hesitant group (AOR = 0.947, *p* < 0.05).

## 4. Discussion

This paper reports on the first nationally representative survey of law enforcement officer hesitancy toward COVID-19 vaccines during the pandemic. While our study data were collected from February 2021 to March 2022 and the pandemic has progressed to different patterns and impact [47], it is still important to analyze these archived data to help address vaccine hesitancy during future iterations of COVID-19 or other pandemics.

We first address the question of the extent of COVID-19 vaccine hesitancy experienced by LEOs. We found that 40% of our national sample of officers were COVID-19 vaccine hesitant; this rate is at the higher end of what has been found for the general population of adults in the US, where rates have ranged from 16% to 46% [8], thus confirming our first hypothesis. Our 40% rate is lower than the COVID-19 vaccine hesitancy rate reported in one other study of LEOs (49%), but that was a convenient sample of police personnel who read a specific law-enforcement magazine [35]. There are reasons why one might expect officers to be less COVID-19 vaccine hesitant than the public. That is, police officers would seem to have greater access to reliable health information from government sources than the general public. Indeed, the COVID-19 vaccine hesitancy rate for LEOs was a bit lower than the rate of vaccine hesitancy reported by firefighters and EMS workers (~52%) [48], frontline personnel who are more likely to be volunteers than the police force [49] and thus perhaps less connected to governmental health information.

Another part of our first research question was whether we would find higher rates of COVID-19 vaccine hesitancy among some demographic subgroups of LEOs. We found some subgroup differences. That is, as seen in prior COVID-19 vaccine hesitancy studies [8], we found in our binomial logistic regression model that officers with higher education (i.e., a college degree or higher) were less likely to be COVID-19 vaccine hesitant compared to officers without a college education. We also observed this same finding in our multinomial regression model for all three vaccine-hesitant groups. Perhaps greater education among some LEOs leads them to a better understanding of the science and safety behind the COVID-19 vaccines. Moreover, as seen in prior COVID-19 vaccine hesitancy studies [8], the older the officer, the less likely he or she is to be COVID-19 vaccine hesitant. We observed this in our binomial logistic regression model and in our multinomial regression model for Groups 2 (will get COVID-19 vaccine when it is proven safe/effective) and 3 (will not get COVID-19 vaccine). This may reflect the common awareness of the greater chance of hospitalization or death for older persons who contract COVID-19 [50]. The current findings appear to point to acceptance of this age-related health-risk information, leading to a greater willingness to receive the COVID-19 vaccine among older officers. While we also expected to observe differences according to biological sex and race/ethnicity, as seen in in prior studies [5,8], we found no statistical differences, except one in the multinomial regression model for Non-Hispanic Black officers being more likely to be in hesitant Group 4 (not sure on receiving a COVID-19 vaccine) than to be in the not-hesitant group compared to non-Hispanic White officers. This may reflect the greater mistrust that some who identify as Black have with the medical field due to historical trauma and experiences of structural discrimination [51,52], which could lead to higher levels of vaccine hesitancy [53].

Our second research question related to the role of looking after your health (went for health checkups and used services of a medical professional) and emotional wellness had in being related to vaccine hesitancy. The prior literature [8] suggests that there would be a relationship [25]. None of these variables proved to be statistically significant in the binomial logistic regression model. In contrast to our expectations based on the literature [8], this sample did not exhibit a positive association between emotional distress and vaccine hesitancy. This lack of statistical significance might have to do with the nature of our sample. More than half of the officers (53.1%) in our study screened positive for experiencing a common mental health disorder (depression or anxiety) and scored a 72.8 out of 100 (with higher scores being associated with more anxiety and depression), suggesting that we did not have enough variability of scores on the MHI-5 to see a relationship with COVID-19 vaccine hesitancy. Given the high pressure on LEOs as first responders to support public safety, the understaffing and long work hours that arose from early retirements and colleagues out sick, and the media attention on police during this study period, one might expect to observe a high degree of anxiety and a more complex association between mental health and vaccine hesitancy than found in other population samples. Further qualitative research would be constructive to understand officers’ perceptions of their experience and choices during that period. Samples with other first responders, who are experiencing less depression and anxiety, might bear a different relationship with COVID-19 vaccine hesitancy.

However, we did find one significant effect in the multinomial regression model. Those who did not report getting a health checkup in the past two years were more likely to be in the hesitant Group 2 (will get COVID-19 vaccine when proven safe/effective) than to be in the not-hesitant group. The reason we did not find more differences could be that most officers were fairly attentive to their health in our sample (e.g., 80% of the study’s LEOs had gone for a health checkup in the past two years, and, on average, most of the study LEOs had seen a medical professional 3.9 times in the past year). The availability of health insurance for full-time sworn officers (compared to access in the general population) may be more of a driver of their healthcare usage than a personal commitment to taking healthcare precautions. Additionally, there may be requirements or guidance from agencies which may also increase LEOs’ use of health services. Alternatively, the misinformation about the COVID-19 vaccine may have overwhelmed officers’ tendency to take care of their health [54].

Our third research question examined whether aspects of the law enforcement officers’ work environment, particularly around COVID-19, would be related to COVID-19 vaccine hesitancy among LEOs. We found that LEOs who were commanders in rank were less likely to be COVID-19 vaccine hesitant compared to line officers. We observed this finding in our binomial logistic regression model and in our multinomial regression model for Group 3 (will not get COVID-19 vaccine). This is a new finding for the field and not one that has been studied before. While years of experience as an officer was only significant for the Group 4 (not sure I will get the COVID-19 vaccine), it does make sense that police personnel at the commander level would be less vaccine hesitant, with this being attributable perhaps to their greater access to more reliable health information from reputable sources. Perhaps commanders have developed greater skills in sorting through complex competing material, a skill they may have refined on the job, to arrive at more science-based judgements such as getting the COVID-19 vaccine. Moreover, commanders may have a greater sense of responsibility for protecting their officer colleagues. We also found that the type of environment the officer worked in made a difference. Officers working in a mixed sector (urban, suburban, and rural areas) were more likely to be in the waiting for a proven safe/effective COVID-19 vaccine than being in the not-hesitant group. Officers working in an “other” sector were more likely to be in the “not sure” group about getting a COVID-19 vaccine than being in the not-hesitant group. Moreover, officers with more years of police experience were less likely to be in this hesitant group of “unsure” than to be in the not-hesitant group.

We had another original finding in our binomial logistic regression model that officers working in agencies that provided no masks for COVID-19 protection were over three times as likely to be COVID-19 vaccine hesitant compared to officers working in agencies that regularly provided masks for COVID-19 protection. As seen in our multinomial regression model, it was Group 3, our most vaccine-resistant group (will not get COVID-19 vaccine), where this finding emerges (no differences for Groups 2 and 4). This is an important factor because it is modifiable, with agencies choosing to provide or not provide masks. While it could be that the agency’s provision of masks was related to the intensity of COVID-19 transmission in a given district, our results are adjusted for urbanicity, and, at times, the virus was blanketing most communities around the United States (e.g., the omicron variant was rampant within our study period) [41]. Alternatively, this finding may be attributable to officers receiving an indirect or unintended message from their agency that COVID-19 was not that serious since they were not being provided a mask and that the COVID-19 vaccine is similarly not particularly important. This correlation highlights the importance of agency policy as an opportunity to encourage officers’ individual health choices.

## 5. Limitations

First, our study relied on cross-sectional data. Therefore, our data can reveal associations but not casual relationships [55]. For example, where we found an association between agencies that provided no masks for COVID-19 protection and COVID-19 vaccine hesitancy, it is not possible to assert a causal relationship. We need additional longitudinal data to address the time-ordering issue. This also plays out for our result that LEOs reported higher vaccine hesitancy compared to aggregated results seen in national general population surveys. It is possible that it is not their status as police officers that is driving these results but rather a third factor related to political ideology. For example, if more officers have conservative political beliefs and conservative political beliefs are associated with greater vaccine hesitancy, it could be the case that the differences that we observe between LEOs and the general public are based on the fact that a high proportion of LEOs are conservative. Therefore, our results have to be viewed cautiously and need to be replicated through additional research.

Next, our study relied on self-reported survey data, thus opening up the potential for recall bias and/or social-desirability bias [56]. However, the collection of survey data with employees is a common accepted practice for collecting reliable data on COVID-19 [5]. Moreover, the surveying of police officers is a very common research methodology [57]. As with other surveys, this study achieved a modest response rate of 30%. However, given the uptick in law enforcement retirements, absenteeism due to the officer’s health or family-member needs, and the expanded emergency service needs during the pandemic, the continued participation of these officers in the OSAW Initiative research was commendable. Furthermore, our study adjusted for non-response by using weights that took into account selection probabilities and addressed possible non-response bias with non-response adjustments. In addition, we promised our sample of officers the confidentiality of their survey responses to promote more honest responses: they were assured (through a legal Privacy Certificate) that their agency would not know they completed a survey nor be provided any information on their specific responses.

Another limitation of our study was in our measures. While there are established scales in this field to measure COVID-19 vaccine hesitancy, such as the WHO COVID-19 Vaccine Hesitancy tool [58] and the Oxford COVID-19 vaccine hesitancy scale [59], we used a tested shorter measure developed by Fisher and colleagues [17] to fit the items into an existing survey designed to measure broader health issues than COVID-19. Future studies with officers should consider adopting these more detailed scales [60]. In addition, we lacked measures on some additional variables that might have been related to COVID-19 vaccine hesitancy. Given that our study was a broader epidemiological study about safety and wellness issues for LEOs, we were limited in the available survey space for additional questions on COVID-19 vaccine hesitancy. However, we believe that future research on COVID-19 vaccine hesitancy among officers should include measures of political affiliation, which have been found to be associated with vaccine hesitancy for healthcare workers [61] and the general population [8]. Future research should look at vaccine history in other areas, such as receipt of influenza vaccination in the previous year. Receipt of influenza vaccination in the previous year was found to be correlated with COVID-19 vaccination hesitancy in a number of studies [8,23,48]. One’s source of COVID-19-related health information (including social media), belief in COVID-19-related conspiracy theories, and people’s negative health beliefs toward COVID-19 vaccines should also be included in future research with LEOs, as these things are related to COVID-19 vaccination hesitancy [8,23]. For example, as suggested by Ruiz and Bell in regard to the general public [23], future research with LEOs should collect more data on their use of social media, traditional media, and news sites and then link their use patterns to their COVID-19 vaccine hesitancy to provide some warnings on common sites for misinformation and how it affects one’s vaccine-hesitancy levels. Other factors that have been found to be related to COVID-19 vaccine hesitancy that should be examined in the future include school closures and religious denomination [8,23].

## 6. Implications for Policy and Practice

This study fills an important gap in public health research, which rarely studies the health of LEOs in the US [62], especially with nationally representative samples [63]. On an issue as global and important as COVID-19 and COVID-19 vaccine hesitancy, this study’s results for LEOs are about the only available results in public health on vaccine hesitancy with direct application for the law enforcement community, and this is the only LEO study on this issue that used nationally representative sampling methods. This study’s results would also potentially have applicability to LEOs who might face other viruses from future pandemics and have to make decisions on receiving new vaccines. Without changes to how the field approaches education for LEOs on vaccines and vaccine hesitancy—with a focus on leadership and administrators to ensure the provision of protective protocols as normative behavior in the agency community—it seems unlikely that the high vaccine hesitancy rates documented in this study would be any different. The public health implications of our work suggest that we should not assume that LEOs will make decisions around vaccination in line with the best scientific information. That is, more direct education on this issue appears to be necessary.

Law enforcement leadership working with public health experts needs to consider opportunities to improve LEOs’ knowledge and education about COVID-19 vaccine safety, effectiveness, illness/side effects, and fears and even enhance law enforcement organizational culture to improve vaccination rates. While public health outreach efforts cannot change an officer’s demographics, outreach has the potential to educate the uninformed; challenge disinformation about future COVID-19 vaccines, as well as vaccines in general; and help those most at risk for serious illness from COVID-19 infection to accurately appraise their personal risk [23]. A systematic review of interventions to address vaccine hesitancy among the general public indicates that approaches have been varied and met with mixed results, but the most successful interventions are multifaceted and dialogue-based ones [64]. Research by Dubov and colleagues with other frontline workers (healthcare workers) found that personal narratives about vaccine safety and efficacy may be more effective for those who are “misinformed”, but motivational interviewing may be better suited to those who are “undecided” [65]. Other interventions that have had some success with healthcare workers should be tested with LEOs, such as educational lectures and leaflet distributions regarding COVID-19 vaccination and arranging testing for allergies to the vaccine [66].

The ongoing COVID-19 pandemic places LEOs, as frontline workers, in continual close personal exposure to the public, many of whom are still testing positive for COVID-19, placing LEOs at a high risk for infection. The COVID-19 vaccine holds great promise as a tool to protect those LEOs from hospitalization or death from COVID-19, but only if they receive the vaccine. Further research is needed to understand what information would be most salient for LEOs to know about vaccine safety and how to convey this information to LEOs with concerns. While we might want to hold professionals such as law enforcement officers to a higher standard when it comes to accepting the COVID-19 vaccine, like healthcare workers with medical training and clinical experience, the same emotions and dilemmas that all members of the general population experience can affect officers and healthcare workers alike [33]. In fact, the officers in our study appeared to be more COVID-19 vaccine hesitant than the general public [8], and this is particularly concerning given that officers are more likely to expose themselves and the community to COVID-19 due to their heavy contact with the public, as part of the routine aspect of their work, thus directly threatening the health of the general public. While no single message or one set of messengers is likely to suffice, our results highlight the heterogeneity in the LEO population in regard to vaccine hesitancy and underscore the importance of tailored messaging to younger LEOs, officers without college degrees, officers with less law enforcement experience, and line officers and supervisors to address their concerns. Prior research suggests that officers are often influenced by agency leadership and policies [67] in terms of their behavior, and this could work with addressing COVID-19 vaccine hesitancy among officers as well.

The COVID-19 vaccines had not been available long enough at the time of our survey data collection to see many mandates. It is possible that a small number of agencies in our study mandated their officers to get the COVID-19 vaccine. An interesting question is how many officers during the pandemic were mandated to be vaccinated. In addition, how many officers followed the mandate, and how many decided to leave their job in opposition to the mandates? Given that many employers at this stage in the pandemic in early 2023 have dropped COVID-19 vaccine mandates, the opportunity to explore this question has diminished for the time being.

## 7. Conclusions

This nationally representative survey of active-duty officers during the COVID-19 pandemic found that 40% of US law enforcement officers were COVID-19 vaccine hesitant. This rate is at the higher end of what has been found for the general population but lower than the rates reported by other front-line workers. We found that officers with higher education, older officers, officers with more law enforcement experience, officers who received recent health checkups, and commanders (compared to line officers) were less likely to be COVID-19 vaccine hesitant. Furthermore, officers working in agencies that provided masks for COVID-19 protection were less likely to be COVID-19 vaccine hesitant (compared to no mask provision). Recognizing that the COVID-19 pandemic remains dynamic in the US and given the rapidly changing public health and political landscape, ongoing research with LEOs is needed. In particular, more research is needed to better shape the messaging around COVID-19 vaccine acceptance for officers and make sure that it is adjusted with the times.

## Figures and Tables

**Table 1:** Descriptive analysis of study variables.

Variable	N	M (SD) or *n* (%)
Age	1104	44.91 (8.87)
Biological sex	1106	
Female		338 (30.6%)
Male		768 (69.4%)
Race/ethnicity	1090	
Non-Hispanic White		803 (73.7%)
Non-Hispanic Black		114 (10.5%)
Hispanic		107 (9.8%)
Other		66 (6.1%)
Education	1098	
Less than a college graduate		507 (46.2%)
College graduate or above		591 (53.8%)
In a relationship	1090	
Married or living with a partner		859 (78.8%)
Other		231 (21.2%)
Hesitancy to COVID-19 vaccine	1074	
No, not hesitant to COVID-19 vaccine		641 (59.7%)
Yes, hesitant to COVID-19 vaccine		433 (40.3%)
Agencies providing masks	1080	
No masks provided		25 (2.3%)
Masks provided sporadically		185 (17.1%)
Yes, masks regularly provided		870 (80.6%)
Confirmed COVID Exposure on the Job	1078	
Yes		540 (50.1%)
No		538 (49.9%)
In the last 2 years, have you had a complete health checkup?	1076	
Yes		873 (81.1%)
No		203 (18.9%)
During the past 12 months, how many times have you seen a medical professional?	1071	3.9 (12.24)
Mental Health Inventory (MHI-5)	1015	
No common mental disorders		476 (46.9%)
Common mental disorders		539 (53.1%)
Emotional Wellness continuous score from 0 to 100 (Mean/SD)		72.8 (17.7)
Length of service (years)	1096	18.2 (9.01)
Rank	1103	
Line officer		627 (56.8%
Supervisor		223 (20.2%)
Commander		203 (18.4%)
Other		50 (4.5%)
Sector type	1043	
Only urban		460 (44.1%)
Only suburban		194 (18.6%)
Only rural		164 (15.7%)
Mix of urban + suburban		58 (5.6%)
Mix of urban, suburban, and rural		78 (7.5%)
All other		89 (8.5%)

**Table 2:** Bivariate results of covariates by COVID-19 vaccine hesitancy status.

Variable	Not Vaccine Hesitant	Vaccine Hesitant	
Categorical/Dichotomous Measures	*n* (%)	*n* (%)	X^2^
Gender			0.04
Female	197 (30.8%)	131 (30.3%)	
Male	442 (69.2%)	302 (69.7%)	
Race/ethnicity			5.96
Non-Hispanic White	474 (74.8%)	320 (74.4%)	
Non-Hispanic Black	70 (11.0%)	35 (8.1%)	
Hispanic	60 (9.5%)	42 (9.8%)	
Other	30 (4.7%)	33 (7.7%)	
Education			18.72 ***
Less than a bachelor’s degree	261 (40.8%)	235 (54.3%)	
College graduate or above	378 (59.2%)	198 (45.7%)	
In a relationship			0.39
Married or living with a partner	509 (79.4%)	337 (77.8%)	
Other	132 (20.6%)	96 (22.2%)	
In the last 2 years, have you had a complete health checkup?			0.11
Yes	520 (81.4%)	348 (80.6%)	
No	119 (18.6%)	84 (19.4%)	
Mental Health Inventory (MHI-5)			0.83
No common mental disorders	290 (47.9%)	182 (44.9%)	
Common mental disorders	316 (52.1%)	223 (55.1%)	
Rank			24.55 ***
Line officer	348 (54.3%)	256 (59.3%)	
Supervisor	115 (17.9%)	104 (24.1%)	
Commander	150 (23.4%)	51 (11.8%)	
Other	28 (4.4%)	21 (4.9%)	
Confirmed COVID exposure on the Job			2.09
Yes	331 (51.7%)	204 (47.2%)	
No	309 (48.3%)	228 (52.8%)	
Agencies providing masks			3.15
No masks provided	10 (1.7%)	13 (3.4%)	
Masks provided sporadically	89 (14.6%)	59 (15.2%)	
Yes, masks regularly provided	510 (83.7%)	315 (81.4%)	
Sector type			10.01
Only urban	289 (46.9%)	157 (39.0%)	
Only suburban	117 (19.0%)	77 (19.1%)	
Only rural	87 (14.1%)	75 (18.6%)	
Mix of urban + suburban	33 (5.4%)	22 (5.5%)	
Mix of urban, suburban, and rural	38 (6.2%)	38 (9.4%)	
All other	52 (8.4%)	34 (8.4%)	
**Continuous measures**	M (*n*)	M (*n*)	F
Age	46.32 (640)	42.93 (433)	0.807
During the past 12 months, how many times have you seen a medical professional?	3.35 (636)	4.74 (431)	7.595 **
Emotional wellness	73.1 (606)	72.19 (405)	0.007
Length of service	19.51 (639)	16.30 (431)	0.863

** *p* < 0.01; *** *p* < 0.001.

**Table 3:** Logistic regression for COVID-19 vaccine hesitancy (*n* = 997 after accounting for missing data on covariates) (hesitant, *n* = 387; and not hesitant, *n* = 610).

	AOR	95% C.I. for AOR	
		Lower	Upper
Biological sex (1 = female, and 0 = male)	0.843	0.592	1.2
Race/ethnicity (reference = non-Hispanic White)			
Non-Hispanic black	1.691	0.938	3.049
Hispanic	0.932	0.545	1.592
Other race	1.71	0.867	3.372
Not cohabitating with a partner (1 = yes, and 0 = no)	1.036	0.7	1.533
No complete health checkup in the past two years (1 = yes, and 0 = no)	1.33	0.913	1.936
Officer rank (reference = line officer)			
Supervisor	1.287	0.897	1.846
Commander	0.583 **	0.387	0.878
Other rank	1.185	0.606	2.318
Number of times seen a medical professional in past year	0.756	0.465	1.229
Agency provided masks (reference = yes, masks regularly provided)			
No masks provided	3.354 *	1.138	9.883
Masks provided sporadically	0.981	0.655	1.467
Sector type where officer works (reference = only urban environment)			
Suburban only	1.163	0.809	1.672
Rural only	0.902	0.576	1.412
Mixed sector	1.446	0.889	2.353
Other sector	1.525	0.881	2.637
Officer has a college degree or higher education (1 = yes, and 0 = no)	0.528 ***	0.393	0.711
Age of officer	0.957 ***	0.932	0.982
Length of service (years an officer)	0.996	0.971	1.021
MHI Score SF36 Scale	1.004	0.996	1.013
Constant	4.603		

* = *p* < 0.05, ** = *p* < 0.01, *** = *p* < 0.001.

**Table 4:** Multinomial logistic regression for COVID-19 vaccine hesitancy (*n* = 997).

(Dependent Variable Ref. = Group 1: Received COVID-19 Vaccine or Will When It Is Available, *n* = 610)	Group 2: Will Get COVID-19 Vaccine When Proven Safe/Effective (*n* = 71)	Group 3: Will Not Get COVID-19 Vaccine (*n* = 240)	Group 4: Not Sure (*n* = 76)
	AOR (95% CI)	AOR (95% CI)	AOR (95% CI)
Biological sex (1 = female, and 0 = male)	1.178 (0.615–2.257)	0.705 (0.464–1.073)	1.117 (0.576–2.163)
Race/ethnicity (reference = non-Hispanic White)			
Non-Hispanic black	1.669 (0.529–5.266)	1.423 (0.694–2.917)	2.757 * (1.061–7.164)
Hispanic	1.378 (0.534–3.558)	0.919 (0.498–1.696)	0.642 (0.188–2.187)
Other race	1.513 (0.411–5.573)	1.464 (0.670–3.195)	3.090 (0.971–9.831)
Not cohabitating with a partner (1 = yes, and 0 = no)	1.221 (0.585–2.548)	1.172 (0.754–1.822)	0.529 (0.222–1.260)
Did not complete health checkup in the past two years (1 = yes, and 0 = no)	2.469 ** (1.298–4.699)	1.142 (0.732–1.782)	1.272 (0.630–2.570)
Officer rank (reference = line officer)			
Supervisor	1.456 (0.736–2.883)	1.307 (0.866–1.973)	0.881 (0.420–1.846)
Commander	0.339 (0.109–1.055)	0.611 * (0.384–0.974)	0.671 (0.297–1.514)
other rank	2.106 (0.639–6.933)	1.106 (0.510–2.396)	0.720 (0.176–2.937)
Number of times seen a medical professional in past year	0.795 (0.336–1.881)	0.545 (0.295–1.008)	1.638 (0.729–3.680)
Agency provided masks (reference = yes, masks regularly provided)			
No masks provided	3.217 (0.653–15.841)	4.117 * (1.306–12.97)	1.312 (0.105–16.379)
Masks provided sporadically	0.920 (0.427–1.983)	1.205 (0.774–1.878)	0.387 (0.135–1.111)
Sector type where officer works (reference = only urban environment)			
Suburban only	0.631 (0.284–1.401)	1.256 (0.835–1.878)	1.322 (0.636–2.748)
Rural only	0.717 (0.298–1.722)	1.109 (0.668–1.841)	0.540 (0.211–1.380)
Mixed sector	2.246 * (1.040–4.852)	1.538 (0.893–2.648)	0.262 (0.052–1.330)
Other sector	0.741 (0.210–2.614)	0.807 (0.382–1.704)	5.067 *** (2.275–11.29)
Officer has a college degree or higher education (1 = yes, and 0 = no)	0.539 * (0.300–0.968)	0.620 ** (0.441–0.871)	0.305 *** (0.167–0.559)
Age of officer	0.922 ** (0.874–0.93)	0.958 ** (0.930–0.988)	0.966 (0.923–1.012)
Length of service (years an officer)	0.994 (0.941–1.050)	1.011 (0.982–1.040)	0.947 * (0.905–0.991)
MHI Score SF36 Scale	1.014 (0.997–1.031)	1.003 (0.993–1.013)	1.003 (0.987–1.019)

* = *p* < 0.05, ** = *p* < 0.01, *** = *p* < 0.001.

## Data Availability

The data are archived with the University of Michigan, ICPSR program under the following title of the project: Officer Safety and Wellness (OSAW) initiative.
